# Epidemic resurgence of dengue fever in Singapore in 2013-2014: A virological and entomological perspective

**DOI:** 10.1186/s12879-016-1606-z

**Published:** 2016-06-17

**Authors:** Hapuarachchige Chanditha Hapuarachchi, Carmen Koo, Jayanthi Rajarethinam, Chee-Seng Chong, Cui Lin, Grace Yap, Lilac Liu, Yee-Ling Lai, Peng Lim Ooi, Jeffery Cutter, Lee-Ching Ng

**Affiliations:** Environmental Health Institute, National Environment Agency, 11, Biopolis Way, #06-05-08, Singapore, 138667 Singapore; Communicable Diseases Division, Ministry of Health, College of Medicine Building, 16 College Road, Singapore, 169854 Singapore; National Public Health Laboratory, Ministry of Health, College of Medicine Building 16 College Road, Singapore, 169854 Singapore; School of Biological Sciences, Nanyang Technological University, 60 Nanyang Drive, Singapore, 637551 Singapore

**Keywords:** Dengue, *Aedes aegypti*, House index, Genotype, Epidemic, Surveillance, Virology, Entomology, Control

## Abstract

**Background:**

Dengue resurged in Singapore during 2013-14, causing an outbreak with unprecedented number of cases in the country. In the present study, we summarise the epidemiological, virological and entomological findings gathered through the dengue surveillance programme and highlight the drivers of the epidemic. We also describe how the surveillance system facilitated the preparedness to moderate epidemic transmission of dengue in the country.

**Methods:**

The case surveillance was based on a mandatory notification system that requires all medical practitioners to report clinically-suspected and laboratory-confirmed cases within 24 hours. The circulating Dengue virus (DENV) populations were monitored through an island wide virus surveillance programme aimed at determining the serotypes and genotypes of circulating virus strains. Entomological surveillance included adult *Aedes* surveillance as well as premise checks for larval breeding.

**Results:**

A switch in the dominant serotype from DENV-2 to DENV-1 in March 2013 signalled a potential spike in cases, and the alert was corroborated by an increase in average *Aedes* house index. The alert triggered preparedness and early response to moderate the impending outbreak. The two-year outbreak led to 22,170 cases in 2013 and 18,338 in 2014, corresponding to an incidence rate of 410.6 and 335.0 per 100,000 population, respectively. DENV-1 was the dominant serotype in 2013 (61.7 %, *n* = 5,071) and 2014 (79.2 %, *n* = 5,226), contributed largely by a newly-introduced DENV-1 genotype III strain. The percentage of houses with *Ae. aegypti* breeding increased significantly (*p* < 0.001) from 2012 (annual average of 0.07 %) to 2013 (annual average of 0.14 %), followed by a drop in 2014 (annual average of 0.10 %). *Aedes* breeding data further showed a wide spread distribution of *Ae. aegypti* in the country that corresponded with the dengue case distribution pattern in 2013 and 2014*.* The adult *Aedes* data from 34 gravitrap sentinel sites revealed that approximately 1/3 of the monitored sites remained at high risk of DENV transmission in 2013.

**Conclusions:**

The culmination of the latest epidemic is likely to be due to a number of demographic, social, virological, entomological, immunological, climatic and ecological factors that contribute to DENV transmission. A multi-pronged approach backed by the epidemiological, virological and entomological understanding paved way to moderate the case burden through an integrated vector management approach.

## Background

Dengue fever is currently the most prevalent mosquito-borne viral disease, especially in the Americas and Asia, caused by Dengue virus (DENV) which is transmitted to humans primarily by *Aedes aegypti* and *Ae. albopictus* mosquitoes [1, 2]. DENV is a positive sense single stranded enveloped RNA virus of the genus *Flavivirus* [3]. Its genome is approximately 11.8 kb in size, and encodes for a single polypeptide flanked by highly structured 5’ and 3’ untranslated regions. DENV exists in four phylogenetically and antigenically distinct serotypes (DENV1-4) and exposure to a particular serotype elicits type-specific life-long immunity [4]. The virus is further subdivided into genotypes based on envelope (*E*) gene and envelope/non-structural protein 1 (*E/NS1*) gene junction regions [5, 6]. These genotypes show a characteristic geographical distribution [7], implying a competitive advantage for individual genotypes that differ in their ability to spread and cause disease in a particular region.

DENV infection causes a clinical syndrome that is primarily benign, but can seldom be fatal due to increased vascular permeability, leading to systemic shock and multi-organ failure [8]. It is estimated to affect approximately 50–200 million individuals each year. More than 125 countries distributed across Americas, Southeast Asia and the Western Pacific regions are endemic to DENV, where almost 50 % of the world population lives at risk [1, 9]. The magnitude of dengue incidence has increased by 30-fold in the past five decades, mainly contributed by rapid urbanization, overcrowding, increased global travel and expansion of vector populations. Except for the tetravalent vaccine (Dengvaxia®) approved recently in a few countries, there is neither an effective antiviral agent nor a licensed vaccine against DENV in many endemic settings where vector control remains as the sole strategy for epidemic control and prevention of dengue.

Dengue fever replaced malaria as the most important mosquito-borne disease in Singapore in the mid-1960s. As a result, a nationwide integrated *Aedes* mosquito control programme was introduced at that time to suppress the vector population and thereby DENV transmission in the country. The programme primarily focused on vector source reduction through surveillance, enforcement, community engagement, careful urban planning and operational research. Consequently, the *Aedes* house index was successfully brought down from about 50 % in 1960s to less than 5 % by the late 1970s. This resulted in a corresponding decline in dengue incidence [10–13]. However, dengue fever started to resurge in the country in 1980s, in a typical 5–6 year epidemic cycle. The magnitude of epidemics escalated within each cycle during the last decade, despite maintaining a consistently low *Aedes* house index (below 1 %) [14–16]. It has been postulated that the resurgence is due to multiple factors that facilitate DENV transmission: an increase in human population density, low herd immunity resulting from long periods of low transmission [17–20], improved local transportation that facilitates virus dissemination, increased travel-related virus importations and the geo-expansion of *Ae. aegypti* in parallel to urban developments. It could also be partly due to improved diagnostic and notification rates over the years. These changing epidemiological settings signaled a need for a strategic revision of the integrated *Aedes* mosquito control programme. Based on programme reviews in the last decade, an enhanced approach for dengue control organised around three key approaches; inter-epidemic surveillance and control, risk based prevention and intervention as well as coordinated intersectoral cooperation, has been introduced. The current integrated surveillance framework is supported by four main pillars; 1). Improved operational response through enhanced case surveillance by using rapid diagnostics; 2). Early warning of outbreaks based on virus surveillance; 3). Understanding the distribution of vectors and their density fluctuations through entomological surveillance; 4). Understanding the relationship between environmental parameters and outbreak risk. Currently, all four serotypes of DENV circulate in Singapore [21] where one serotype is typically dominated for a particular period until it is replaced by another serotype. Such “serotype switch” events have preceded previous epidemics [15]. A switch from DENV-2 to DENV-1 in early 2004 was associated with an outbreak during 2004-2005. In early 2007, DENV-2 gained dominance from DENV-1 and drove the 2007-08 epidemic of over 8,000 cases [15]. Dengue incidence stabilized subsequently around an annual figure of 5,000 cases until the end of 2012. Singapore experienced yet another unprecedented increase in dengue cases in 2013 and 2014, recording 22,170 and 18,338 cases, respectively [22].

In the present study, we present an account of the resurgence of dengue fever in Singapore during 2013-14, the worst known dengue fever encounter in the country. We describe how the surveillance system led to an early warning of the outbreak, and how it facilitated preparedness within the country. We also present the virological and entomological findings gathered through the surveillance programme.

## Results

### Early warning of an impending outbreak

In March 2013, the proportion of DENV-1 rose from 20–30 % to more than 50 %, replacing DENV-2 as the predominant serotype. The switch in predominant serotype signaled a potential spike in cases in the approaching “dengue season” during May-August. The alert was further corroborated by the earlier than usual escalation of weekly cases at the beginning of 2013, and an increase in average *Aedes* house index from 0.17 (range: 0.12–0.27) in 2012 to 0.19 (range: 0.11–0.25) in 2013. In epidemiological week 9 (March 2013), a newly-developed statistical model [23] provided a forecast of about 800 cases per week at the peak of the epidemic in June. The temporal model utilizes data obtained through case and vector surveillance as well as weather parameters [23]. Based on the multiple warning signs, the National Environment Agency (NEA) enhanced its vector control in the community and initiated preparation to respond to the outbreak. All stakeholders were also warned through the Inter-Agency Dengue Task Force [14] of the impending epidemic risk. The community was galvanized through a campaign in April 2013.

### Epidemiological findings

**Fig. 1:**
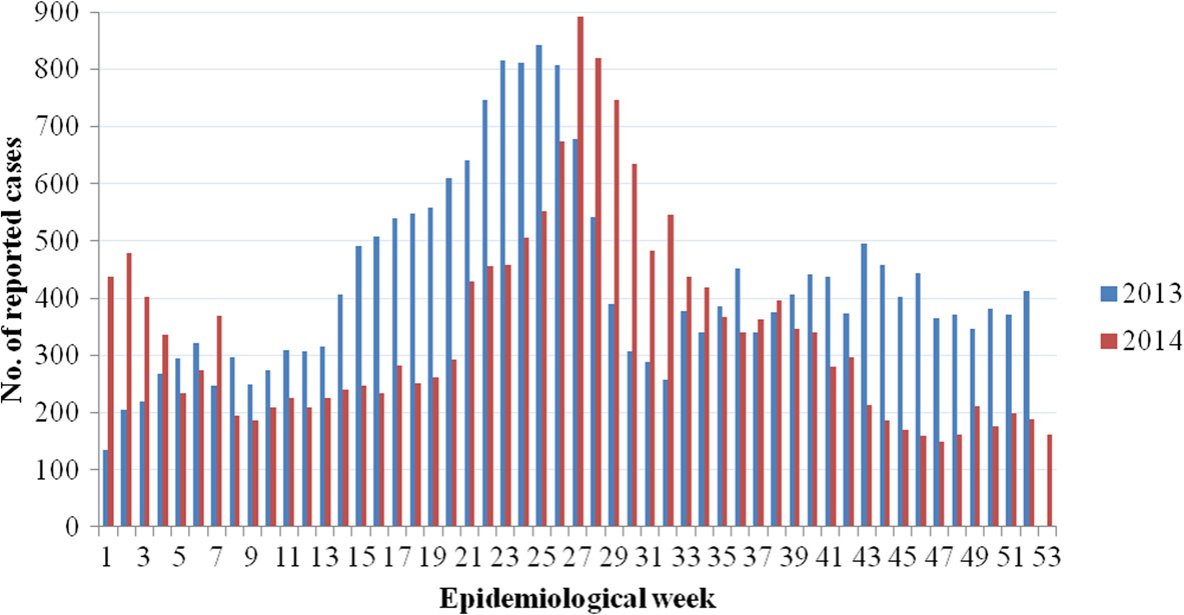
Epidemiological curve for weekly dengue cases in 2013 and 2014. All cases are laboratory confirmed

The epidemic took off in January 2013 (week 1–5) and weekly cases showed a rapid surge in April (week 14–18) to reach the first peak in June 2013 (week 23–26) (Fig. 1). There were 134 cases in the first week of January 2013, which escalated to reach 842 cases in 25^th^ week. The trend declined and stabilized between September 2013 (week 36–39) and January 2014 (week 1–5). The second peak was observed in July 2014, reaching 891 cases in 27^th^ week, the highest weekly number of dengue cases ever recorded in Singapore. The epidemic started to subside in November 2014 (week 45–48), recording the lowest number of weekly cases in 47^th^ week (*n* = 149).

The total number of laboratory-confirmed cases reported in 2013 (*n* = 22,170) and 2014 (*n* = 18,338) corresponded to an incidence rate of 410.6 and 335.0 per 100,000 population, respectively. The incidence rate in 2013 was the highest reported so far in Singapore and exceeded the previous highest recorded in 2005 (333.1 per 100,000 population, *n* = 14,210). The diagnostic rate at Environmental Health Institute (EHI) diagnostics surged from 27 % (*n* = 86) of the total number of samples tested in January 2013 to 41 % (*n* = 139) in March, 12 weeks ahead of the first peak (Fig. 1). The rate dropped gradually and maintained between 20–30 % from July 2013 to reach the lowest (15 %) in February 2014. An upward momentum occurred subsequently to reach the peak diagnostic rate (46 %) in April followed by another peak (49 %) in July 2014. The second peak coincided with the highest number of weekly cases in 2014. The relationship between the EHI diagnostic rate and total number of reported cases from 2012 to 2014 showed a positive correlation (Spearman’s correlation =0.73, *P* < 0.05).

### Virological findings

**Fig. 2:**
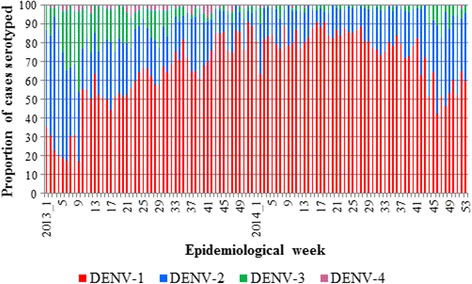
Weekly distribution of DENV serotypes in Singapore: 2013-2014. The serotype proportions were calculated based on 14,814 patient sera successfully typed in 2013 (*n* = 8,216) and 2014 (*n* = 6,598)

DENV serotypes were confirmed in 8,216 samples in 2013 and 6,598 samples in 2014, which corresponded to 37.1 and 36.0 % coverage of all dengue cases reported in respective years. These viruses also included 45 and 12 specimens of *Ae. aegypti* and *Ae. albopictus* collected in 2013 and 2014, respectively. In 2013, the overall serotype composition was dominated by DENV-1 (61.7 %, *n* = 5,071), followed by DENV-2 (24.8 %, *n* = 2,034), DENV-3 (11.5 %, *n* = 941) and DENV-4 (2.0 %, *n* = 170). The trend continued in 2014; DENV-1 (79.2 %, *n* = 5,226), DENV-2 (18.1 %, *n* = 1,194), DENV-3 (2.5 %, *n* = 165) and DENV-4 (0.2 %, *n* = 13). As shown in Fig. 2, the serotype composition was more diverse in 2013, especially during the first half of the year, than in 2014, suggesting that a mixed viral population circulated during the early epidemic establishment period.

A total of 1,270 complete *E* gene sequences was generated in 2013 (Table 1). The corresponding number for 2014 was 1,531. The sequence collection included 38 mosquito-derived virus sequences in 2013 and another six sequences detected among mosquitoes trapped in 2014. The overall genotype coverage was 4.5 % and 8.4 % of all reported cases in 2013 and 2014, respectively. The majority (60.6 %, *n* = 770) of virus strains sequenced in 2013 were DENV-1, followed by DENV-2 (26.1 %, *n* = 332) (Table 1). Similarly, DENV-1 and DENV-2 constituted 75 % (*n* = 1150) and 21.8 % (*n* = 334) of genotyped samples in 2014, respectively. The proportion of DENV-3 sequences generated in 2013 (*n* = 133, 10.5 %) was 3.6 times higher than that in 2014 (*n* = 43, 2.9 %).

**Table 1:** Composition of DENV genotypes circulated during the epidemic in 2013 and 2014

Serotype	Genotypes/clades	2013	2014
DENV-1	Genotype I	97 (7.6 %)	136 (8.9 %)
	Genotype II	04 (0.3 %)	0 (0 %)
	Genotype III^a^	669 (52.7 %)	1,014 (66.2 %)
DENV-2	Asian I	0 (0 %)	01 (0.1 %)
	Cosmopolitan	02 (0.2 %)	12 (0.8 %)
	Cosmopolitan Clade I	28 (2.2 %)	11 (0.7 %)
	Cosmopolitan Clade 1a	151 (11.9 %)	13 (0.8 %)
	Cosmopolitan Clade 1b	33 (2.6 %)	269 (17.6 %)
	Cosmopolitan Clade III	74 (5.8 %)	0 (0 %)
	Cosmopolitan Clade V	12 (0.9 %)	0 (0 %)
	Cosmopolitan Indian	32 (2.5 %)	28 (1.8 %)
DENV-3	Genotype I	02 (0.2 %)	26 (1.7 %)
	Genotype II	03 (0.2 %)	01 (0.1 %)
	Genotype III	128 (10.1 %)	16 (1 %)
DENV-4	Genotype I	01 (0.1 %)	01 (0.1 %)
	Genotype II	34 (2.7 %)	03 (0.2 %)
Total		1,270	1,531

^a^includes epidemic strains in 2013 (*n* = 666) and 2014 (*n* = 1,010)

The proportion of each genotype among all genotyped cases for the respective year is shown within brackets. The phylogeny of genotypes and clades of DENV serotypes is given in Fig. 4a and b

**Fig. 3:**
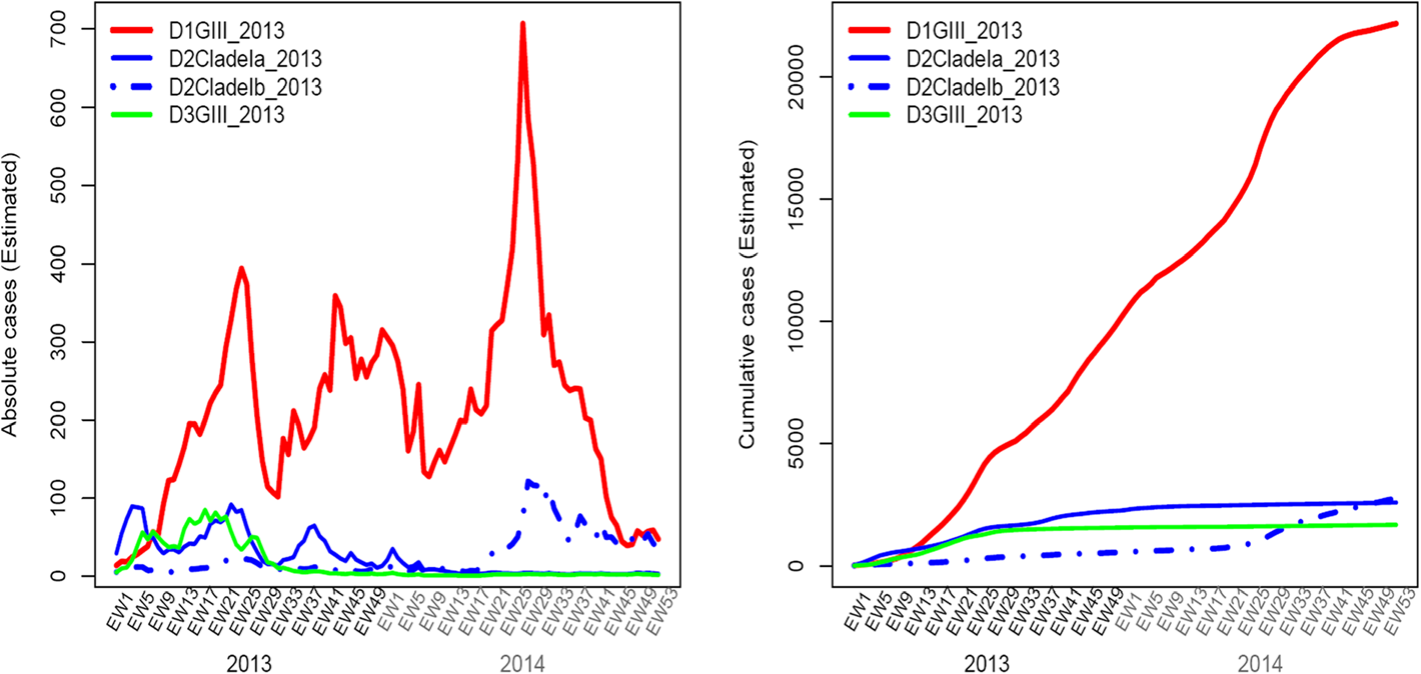
Cumulative dynamics of cases due to the most common DENV strains detected during the epidemic and their case contributory pattern in 2013 and 2014. The weekly national serotype data and weekly EHI genotype data from 2007 to 2014 were used to estimate the historical genotype proportions. In order to obtain smooth estimates of genotype proportions over time, a Bayesian approach was used assuming multinomial distribution of serotypes and genotypes, and an auto-correlated prior distribution for logarithm transformed proportions. Bayesian estimates of the weekly genotype proportions were sampled from the posterior distribution, which were used together with weekly national case count to calculate the weekly cases attributed to each genotype as well the cumulative case count. The analysis was done using R software version 3.1.1 [33]. Only the genotypes dominant during the epidemic years have been plotted in the graph

**Fig. 4:**
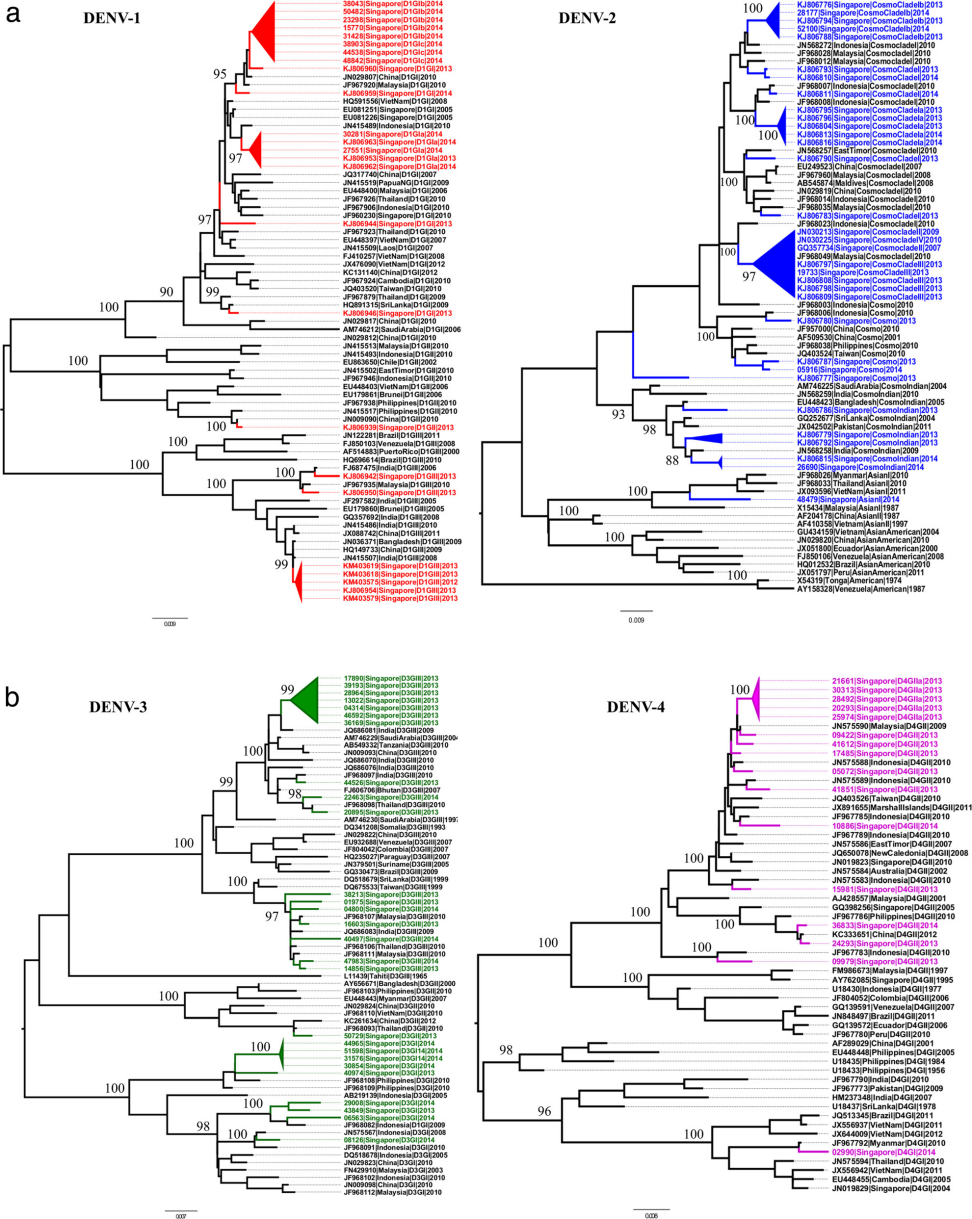
*E*-gene based phylogeny of DENV illustrating different types of virus strains circulated during 2013-2014. Phylogenetic analysis was performed in MEGA6 program [32] using the maximum-likelihood method based on the general time reversible model with gamma distribution and invariant sites. The robustness of the original tree was tested with 1000 bootstrap replications. **a**. DENV-1 and DENV-2 strains circulated in Singapore in both years have been highlighted in red and blue respectively. **b**. DENV-3 and DENV-4 strains circulated in Singapore in both years have been highlighted in green and purple respectively. The major groups summarized in Table 1 are shown in triangular cartoons. Each taxon is named with sample ID/NCBI accession number, reported year, country and genotype information. Numbers on branches are bootstrap support values. GI, GII, and GIII = genotypes I, II and III; cosmo = cosmopolitan genotype

Phylogenetic analysis revealed that DENV-1 genotype III was the dominant genotype among all four serotypes sequenced in 2013 (*n* = 666, 52.4 %) and 2014 (*n* = 1,010, 65.9 %). This “epidemic strain” was first detected in a sporadic case in November 2012, at a time when DENV-2 cosmopolitan genotype was leading the transmission. In January 2013, the epidemic strain of DENV-1 genotype III was detected in a major dengue cluster in the Southeastern part of Singapore, indicating the establishment of indigenous transmission of this strain. It took the lead from DENV-2 cosmopolitan genotype in March 2013 and continued to expand throughout the country. The epidemic strains showed a rapid accumulation rate (Fig. 3) and formed a lineage distinct from those reported earlier locally [21], with 99 % bootstrap support (Fig. 4a). This genetic distinction of the epidemic lineage suggested that its appearance in the country was due to an introduction. Its closest relatives were DENV-1 genotype III strains reported from India, Bangladesh and China during 2008-2011 periods (Fig. 4a), indicating the circulation of genetically-related virus strains in Asia preceding the epidemic and thereby the likelihood of its introduction from a regional country.

In addition, the early establishment phase of the epidemic saw an uprising of two new strains: DENV-2 cosmopolitan genotype clade I (named as clade Ia) and DENV-3 genotype III (Fig. 4a and b). Both strains formed genetically distinct clusters in respective phylogenies (Fig. 4a and b) and were likely to be newly introduced. Clade Ia shared ancestry with DENV-2 strains reported earlier from Indonesia during 2010-2012 periods, whereas DENV-3 genotype III strains were related to those reported from India during 2009-2010 periods. The first evidence of DENV-2 cosmopolitan clade Ia in Singapore was in June 2012 (23^rd^ week). Prior to the setting out of the epidemic, clade Ia strains maintained a low profile, but showed a rapid surge in case accumulation from January 2013 and started to subside at the end of the year. There were only 30 clade Ia strains in 2012, whereas 151 sequences were generated in 2013. On the other hand, DENV-3 genotype III strains emerged in January 2013, at the beginning of the epidemic. Its circulation lasted until 42^nd^ week of 2013. These observations suggested that prevailing favourable conditions for virus transmission during the early phase of the epidemic allowed multiple newly-introduced virus strains to proliferate and spread.

As summarised in Table 1, besides the major strains described above, multiple virus strains belonging to all four DENV serotypes circulated during the epidemic period. The phylogeny representative of all these strains has been illustrated under respective serotypes (Fig. 4a and b). The temporal fluctuation of proportions of each strain among all genotyped cases demonstrated a highly heterogeneous DENV population at any given time, though a few strains were dominant. Those dominant strains were widely distributed across the country, and contributed to 70–80 % of cases during 2013-14. On the other hand, the “minor” strains were less well established and their transmission was generally localized. One of the notable observations was that a relatively higher number of genetically distinct strains, including “minor” strains, circulated in substantial proportions in 2013 than in 2014 (Table 1). Besides the availability of a susceptible human pool, the sustainability of vector-borne virus transmission is affected by the vector density. When the mosquito population size is small, the dominant strains are likely to saturate the infectious mosquito pool relatively fast, leaving little opportunity for “minor” strains to settle down. On the other hand, when the mosquito population expands, especially during peak periods of transmission, the chances for more virus strains to establish transmission also increase. As the control measures are targeted to suppress the *Aedes* population, their numbers fluctuate and virus populations are expected to go through bottlenecks during periods of low *Aedes* density. Importantly, the vector density fluctuations are not universal across the country as areas with higher number of disease clusters, especially major clusters, require more effort and time to bring down the mosquito density than in areas with localized transmission. Consequently, the effects of vector control efforts are more likely to be profound on the “minor” strains that show localized transmission than dominant strains. These findings suggested that favourable conditions for virus transmission prevailed in 2013, but sustained disease control measures may have facilitated the elimination of many DENV strains in 2014. It is noteworthy that immune pressure and evolutionary forces may also have affected the survival of virus strains and may have contributed to fluctuations in virus population independent of vector density.

Nevertheless, disappearance of major strains provided the opportunity for less-common strains to dominate. One such example is the replacement of DENV-2 cosmopolitan clade Ia by clade Ib strains in April 2014. Clade Ib has been circulating since February 2013, but was unable to dominate over clade Ia until the latter became gradually extinct in early 2014. Interestingly, clade Ib was the dominant strain circulated in Johor and Melaka, the southern states of Malaysia, in 2013 [22]. Johor is the closest Malaysian state to Singapore. The detection of identical clade Ib strains in Singapore indicated the possibility of virus exchange between the two countries [22]. Clade Ib’s dominance in Malaysian states also suggested its outbreak potential. In fact, clade Ib has been the dominant DENV-2 strain in Singapore since May 2014.

### Entomological findings

**Fig. 5:**
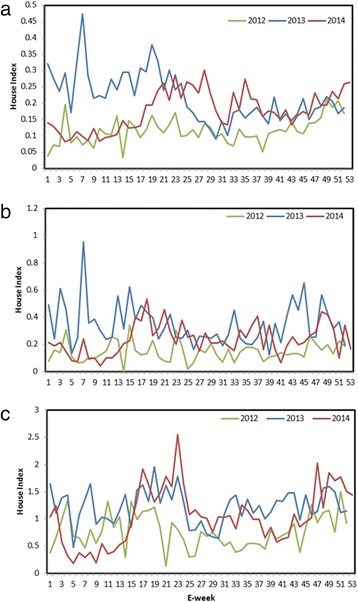
Temporal variation of House Index in different types of residential premises in 2013 and 2014. **a**. Housing Development Board (HDB) apartments, **b**. Private apartments/condominiums and **c**. Landed properties. House Index for each residential premise type is defined as the number of houses detected with *Aedes* mosquito breeding per 100 houses inspected. The analysis is based on the outcome of routine inspections carried out by approximately 800 ground officers from the Environmental Public Health Operations, NEA. *Aedes* immatures were morphologically identified to the species level at EHI. E-week = epidemiological week

The house index for *Aedes* mosquitoes in three main types of residential premises, namely government-sponsored Housing Development Board (HDB) flats, private apartments and condominiums as well as landed properties, increased gradually from the end of September 2012 (Fig. 5). The increase was most obvious in HDB and landed properties categories. Among the *Aedes* immatures collected, the proportion of *Ae. aegypti* mosquito breeding*,* the primary vector of DENV in Singapore, in residential premises was 59.5 and 54.6 % in 2013 and 2014, respectively.

**Fig. 6:**
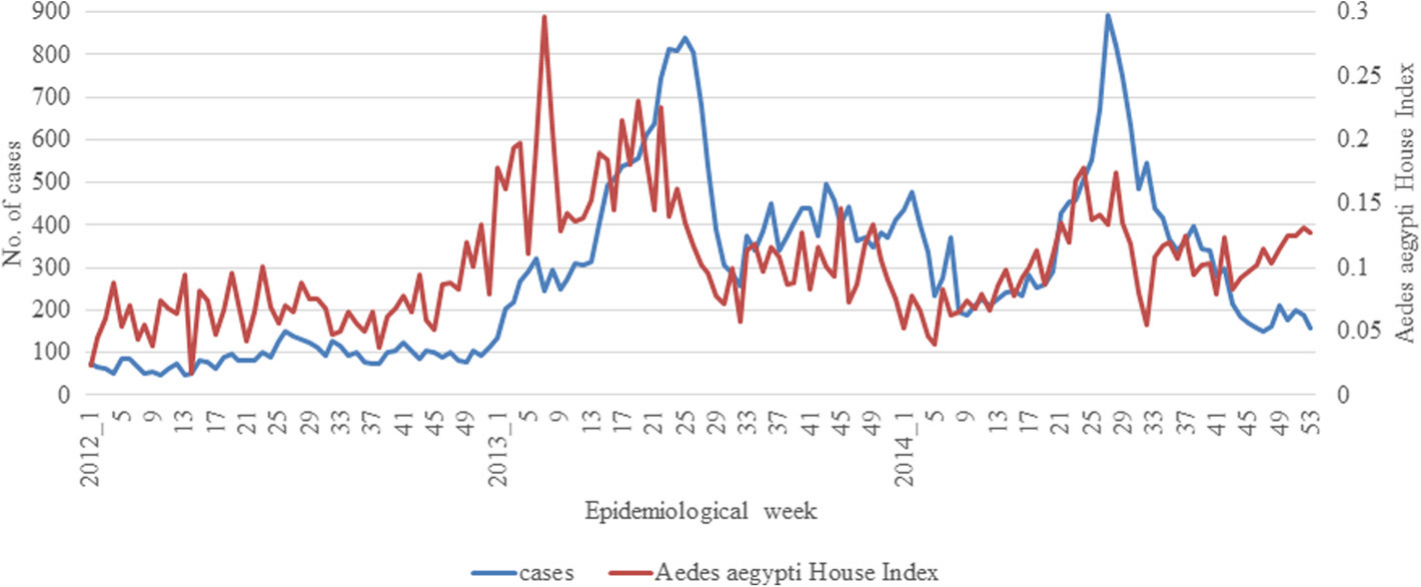
Weekly trends of cases and *Ae. aegypti* house index during 2012-2014. The *Ae. aegypti* house index is expressed as the percentage of houses with *Ae. aegypti* mosquito breeding. The comparison shows that the fluctuation of *Ae. aegypti* house index generally preceded that of cases

**Fig. 7:**
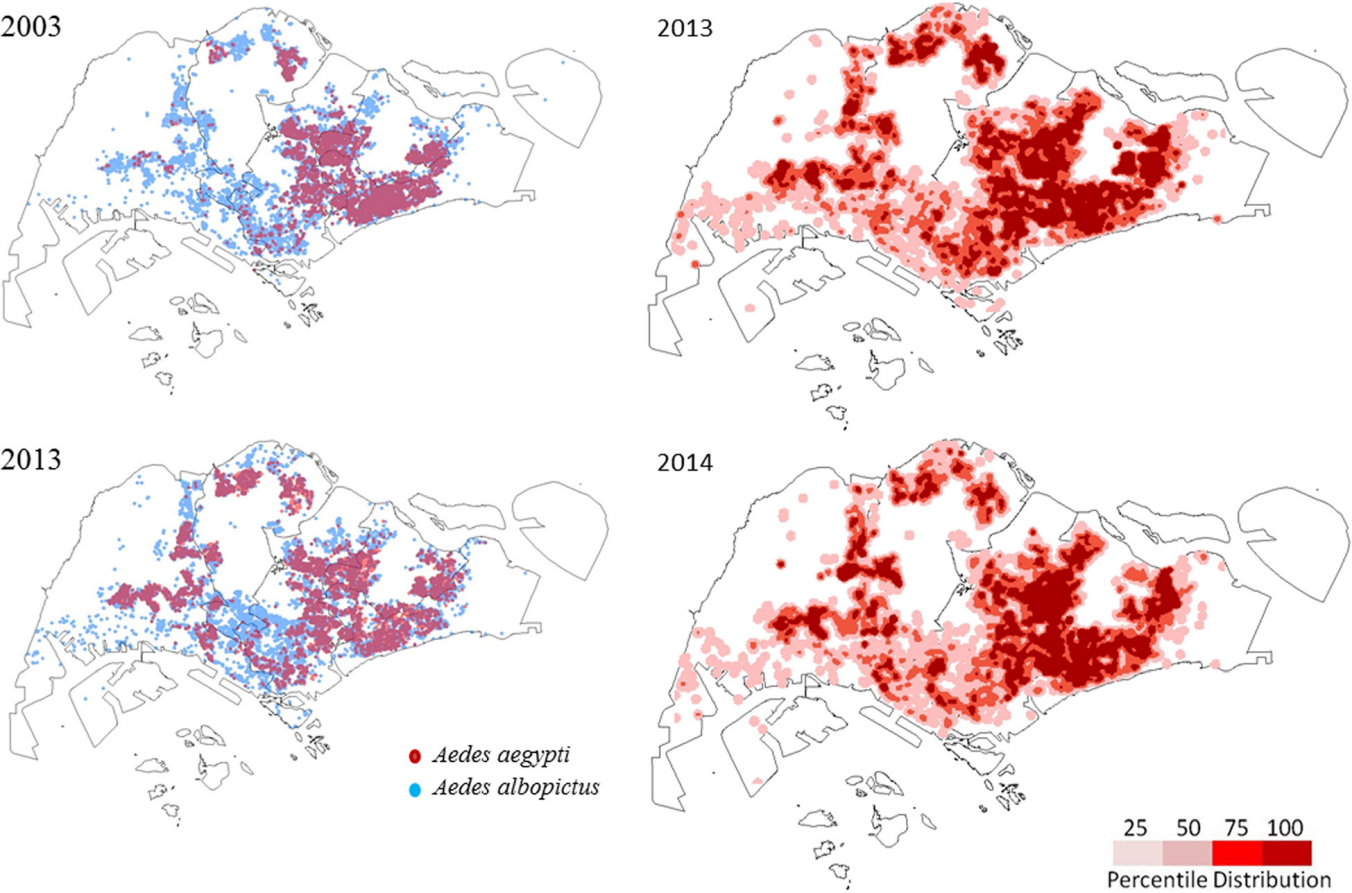
Spatial distribution of *Ae. aegypti* and dengue cases in 2013 and 2014. **a**. Spatial expansion of *Ae. aegypti* breeding in 2013 as compared to 2003. The breeding sites were identified based on island wide *Aedes* breeding data collected on a daily basis as part of vector control operations. The map was generated using the ArcGIS 10.1 ArcMap software (ESRI, CA, USA). **b**. Spatial distribution of dengue cases in 2013 and 2014. The spatial distribution of dengue cases in Singapore was generated using the kernel density tool in the spatial analyst toolbox of ArcGIS 10.1 ArcMap software (ESRI, CA, USA) based on a search radius of 400 m. Case density values were classified into four classes of < 25^th^ (2 cases/km^2^), 25^th^-50^th^ (16-25 cases/km^2^), 51^st^-75^th^ (56-61 cases/km^2^) and more than 75^th^ (217 cases/km^2)^ quantiles, using the quantile classification method and were displayed in tones of pink as shown in the legend

The percentage of houses with *Ae. aegypti* mosquito breeding (named as *Ae. aegypti* house index) was significantly higher (*p* < 0.001) in 2013 (annual average of 0.14) than in 2012 (annual average of 0.07). The index reached its peak at the beginning of the inclination of cases in February 2013 (Fig. 6). The trend continued in 2014 though at a lower magnitude (annual average of 0.10) than in 2013 (Fig. 6). Moreover, the distribution of *Ae. aegypti* expanded dramatically during the decade from 2003 to 2013 in parallel to infrastructure development in the country (Fig. 7a). The new areas generally represented newly-developed urban residential sites that are mainly distributed in south-central and western parts of Singapore (Fig. 7a). As expected, the dengue case distribution pattern in 2013 and 2014 was in line with the geographical spread of *Ae. aegypti* in the country (Fig. 7b).

In the third quarter of 2013, an adult *Aedes* sentinel surveillance system was established with about 3,000 Gravitraps in 34 locations throughout the island, in addition to Gravitraps that were deployed in *ad hoc* cluster management [24]. The surveillance showed that the average Gravitrap *aegypti* index, which expresses the percentage of Gravitraps that caught at least one adult *Ae. aeygpti* in a particular location, was 9.7 % (±0.3 % SE) in 2013. The index declined to 6.2 % (±0.1 % SE) in 2014. Furthermore, the index indicated that 29.4 % of locations (10 of 34) were at high risk of transmission (index ≥ 12.0 % based on unpublished data, EHI) in 2013 and declined to 8.8 % (3 of 34) in 2014.

## Discussion

### Drivers of the epidemic

Case burden of the dengue epidemic in 2013 and 2014 was unprecedented, recording 1.7 times more cases than those reported during the last DENV-1 epidemic during 2004-05. The culmination of the latest epidemic is likely to be due to a number of demographic, social, virological, entomological, immunological, climatic and ecological factors that contribute to DENV transmission.

The most obvious driving force of the epidemic was emergence of a new strain of DENV-1 genotype III. In average, this epidemic strain contributed to 68.5 % of cases among all genotyped cases (*n* = 2,803) during the 2013-14 periods. Serotype data that provided even a higher coverage of reported cases (an average of 36.6 % in both years) confirmed the dominance of DENV-1 during the same period (in average, 69.5 % of serotyped cases). The peak of the epidemic during May-June 2013 was preceded by a change in the dominant serotype from DENV-2 to DENV-1, approximately four months after the first evidence of epidemic strains in November 2012. A similar event has occurred in Singapore during a dengue epidemic during 2007-08, which anchored the phenomenon of “serotype switch” as a warning sign of epidemic risk in the country [15].

Besides highly-potential virus strains, the epidemic transmission of DENV requires the connectivity between two important contributors: a susceptible human population and an optimal vector density. The latter is partially affected by climatic and ecological factors. Once exposed to a particular serotype of DENV, an individual elicits type-specific life-long immunity, but remains immunologically susceptible to a heterotypic DENV infection [4]. Therefore, the long term absence or low prevalence of a particular serotype in an area increases the proportion of population susceptible to infections by the respective serotype. This may explain the dominant emergence of DENV-1 after eight years in Singapore. The last DENV-1 epidemic in the country was in 2005 [14]. The lower prevalence of antibodies reactive to DENV-1 than that of DENV-2 across multiple age groups in a cross-sectional study among healthy individuals aged 16–60 years during 2009-10 also testified that a higher proportion of individuals, especially those within the age group of 16–30 years, remains susceptible to DENV-1 than DENV-2 [20].

Furthermore, the seroprevalence data suggests that strict disease control measures since early 1970’s have widened the susceptible age range by reducing the risk of virus exposure. There has been a gradual reduction in dengue seropositivity from 1982 to 2009 across all age groups [20]. The age-standardized seroprevalence in adults aged 18–79 years dropped from 63.1 % in 2004 to 54.4 % in 2010 [17]. Importantly, the sharpest reduction in seropositivity was seen in less than 20 years old age group, indicating the low level of exposure to dengue among young generations. The overall seroprevalence among individuals aged 16–25 years was 16.1 % during 2009-10 periods [20]. Moreover, the rapid growth of non-resident population (from 19 % in 2005 to 29 % in 2014) in the country due to its reliance on foreign labor is also likely to have contributed to the expansion of susceptible proportion. However, recent data demonstrating a reducing trend of seroprevalence among Singapore resident population [20] and a low level (0.01; 95 % CI: 0.0085–0.012) of the force of infection since 1993 (unpublished data, EHI) suggests that the gradual increase in magnitude of dengue case burden could partially be contributed by the improved rate of case detection in recent years.

The entomological surveillance data indicated an increase in both larval and adult *Ae. aegypti* population during the epidemic period. The average percentage of houses with *Ae. aegypti* mosquito breeding doubled in 2013 as compared to 2012. Moreover, the data suggested *Ae. aegypti* breeding in a high proportion of residential premises in the latest epidemic years. *Aedes aegypti* adult surveillance with Gravitraps also demonstrated high vector density in many locations prior to and during the epidemic. These observations supported the notion that prevailing vector and host factors were favourable for virus transmission at the time of introduction of a new virus lineage and explained its rapid establishment and spread across the island. The ground conditions were conducive to sustain virus transmission on a longer term causing an unprecedented epidemic.

### Epidemic response

In Singapore, the control of vector-borne diseases, including dengue, is carried out by NEA, and the clinical management and surveillance is overseen by the Ministry of Health (MOH) [25]. The close coordination between the two sectors is therefore imperative to tackle vector-borne diseases. While surveillance of vector-borne diseases rides on the national disease surveillance programme at MOH, daily communication of surveillance data enables prompt vector control response by NEA. Therefore, several activities were jointly initiated by the MOH and NEA to manage the epidemic crisis.

1). *Enhanced case surveillance measures to increase the diagnostic coverage and to enable prompt vector control response.* Due to the unprecedented increase in cases during the early phase of the epidemic, the medical community was appraised through MOH to have a high clinical suspicion of dengue among febrile cases. Moreover, an existing network of general practitioners was encouraged to utilize a subsidized laboratory testing service provided by EHI, one of the public health laboratories under NEA, Singapore. 

2). *Projection of case numbers to facilitate stockpiling of diagnostic reagents and to accommodate increased demand on the healthcare system.* Based on the statistical model projection on weekly case numbers [23], arrangements were made to extend EHI diagnostic services during weekends and to stockpile diagnostic reagents several months ahead of the peak of the epidemic. The close communication between MOH and NEA on the weekly case projection also allowed hospitals to plan for increased demand on the healthcare system resulting from extra consultations and admissions. 

3). *Expansion of virus surveillance activities to facilitate the resource allocation for targeted vector control.* In order to achieve a better serotype and genotype coverage during the epidemic, the existing virus surveillance programme at EHI [15, 21] was extended to include samples from polyclinics, hospitals and private laboratories through a joint initiative between NEA and MOH. During non-epidemic periods, the programme generally screens a relatively small proportion of reported cases received through an island wide general practitioner network as an assessment of impending epidemic risk [15, 21]. Consequently, serotype analysis was completed in more than 1/3 of total reported cases and the genotype coverage was doubled from 2013 to 2014. Assisted by geographical information system (GIS)-based plotting of patients’ residential locations, the serotype and genotype data provided a detailed understanding of the spatial and temporal distribution of virus strains within the country. Genetic fingerprinting of virus strains allowed monitoring of their spread between locations and facilitated targeted vector control in newly-introduced sites. The same approach was used to monitor the successful elimination of virus transmission in disease clusters. 

4). *Early launch of the dengue campaign to promote community awareness of dengue situation and prevention of transmission.* As the epidemic was looming, NEA brought forward the launch of “Do the Mozzie Wipeout” campaign to April in 2013. The campaign, which is usually launched at the beginning of “dengue season” in the middle of each year, is part of the community outreach programme aimed at promoting awareness of dengue situation and inspiring action to prevent dengue. A colour-coded alert system was also launched through the Dengue Community Alert System to keep residents informed about active disease clusters in respective localities. The banners coded green (no dengue clusters), yellow (dengue cluster with < 10 cases) and red (dengue cluster with ≥ 10 cases) were displayed to indicate the seriousness of dengue situation in each vicinity. More than 400 individuals were trained as “dengue volunteers” in order to support the community outreach activities that continued throughout the epidemic through seminars, talks, roadshows and media. 

5). *Enhanced source reduction for mosquito breeding through an accelerated premise inspection programme.* The entomological surveillance and enforcement activities were also carried out in parallel. House-to-house checks were conducted by about 800 ground officers who targeted to check every home and surrounding areas for mosquito breeding. Apart from homes, daily checks also cover common ground areas, public areas and congregation areas for potential mosquito breeding spots. The inspection frequency was accelerated to cover all premises within the boundary of disease clusters during the epidemic period. A total of 8,781,935 checks was conducted in residential houses during 2013-14. Chemical larviciding activities were carried out in housing estates, industrial premises and public places on a regular basis and whenever *Aedes* breeding was detected through inspections. The public was encouraged to use pellets containing *Bacillus thuringiensis* in places such as roof gutters. Fogging activities were conducted in major clusters during the peak periods of transmission to reduce adult *Aedes* population density especially when the intensity of transmission sustained despite other source reduction efforts. Data on *Aedes* immatures collected through field inspections was used to gauge *Aedes* population density in a particular area. 

6). *Launch of gravitrap surveillance to monitor the fluctuations of adult Aedes population.* The existing vector population assessment based on *Aedes* breeding data was complemented by adult *Aedes* monitoring with Gravitraps in disease clusters as well as in 34 sentinel locations. 

7). *Guiding resource allocation for targeted vector control based on a spatial risk map.* An island wide risk map developed based on data pertaining to *Aedes* breeding, past dengue exposure, population density, circulating virus strains etc. was used to guide resource allocation for targeted vector control during the epidemic. 

8). *Integrated vector management activities aligned with the whole-of-government’s effort.* Integrated vector management activities in Singapore are closely aligned with the whole-of-government’s effort in establishing people, public and private (3P) partnership to develop innovative and sustainable initiatives to promote environmental ownership in the community. NEA leads an Inter-Agency Dengue Task Force [14] comprising 27 stakeholders from the 3P sectors to coordinate nationwide dengue control efforts. The Inter-Agency Dengue Task Force assisted the epidemic control by ensuring the planning and implementation of operational activities of each agency to align with source reduction and vector control efforts carried out by NEA.

All those activities were aimed at gaining a clear picture of case burden and *Aedes* population over time in order to monitor the intensity and the potential sustainability of disease transmission. In addition to NEA’s source reduction efforts on the ground, the control programme relied on the community participation to minimize human-vector contact and *Aedes* breeding at residential premises. Even though it was difficult to directly measure the impact of those activities on the disease transmission, a comparison between the weekly trends of *Aedes* house index and case data showed that cases generally fluctuated in parallel to mosquito population variations (Fig. 6). The intense control efforts could bring down the case burden steeply from the peaks through the suppression of vector population (Fig. 6).

## Conclusions

The epidemic resurgence of dengue fever in Singapore in 2013 was multi-factorial. The emergence of a new strain of DENV-1 genotype III, expansion of the susceptible human population, favourable conditions for mosquito breeding and a widely-distributed vector population collectively contributed to sustain the virus transmission during the epidemic. A multi-pronged approach backed by the epidemiological, virological and entomological understanding facilitated the resource planning and community awareness that paved way to moderate dengue transmission through an integrated vector management approach.

## Methods

### Case data collection

It is mandatory for medical practitioners and clinical laboratories to notify all clinically-suspected and laboratory-confirmed dengue cases and deaths to MOH within 24 hours of detection. The notification information included demographic data, travel history, clinical data, dates of onset of illness and diagnosis. Notified cases were investigated to determine whether the infections were autochthonous or imported. Patients with no travel history to a dengue endemic area outside of Singapore within the seven days prior to onset of illness were defined as autochthonous cases. Any epidemiological link among cases was established whenever possible. The laboratory confirmation of clinically-suspected cases is achieved either through NS1 antigen detection or viral RNA detection by PCR [26, 27]. Only the laboratory-confirmed cases are officially reported.

Details of cases were sent promptly to NEA to determine the clustering of cases and to conduct site visits for further investigations to aid vector control operations. A dengue cluster is defined as two or more cases epidemiologically linked by place (residential or workplace/school) within a radius of 150 meters and with their onset of illnesses within a 14 day period. Information was uploaded onto the Geographical Information Systems (GIS) server in order to identify areas of active dengue transmission.

### Virological data collection

The circulating DENV populations were monitored through a virus surveillance programme jointly conducted by the MOH and NEA. Briefly, blood samples from suspected dengue patients who sought treatment at general practitioners, public/private hospitals and polyclinics were tested for the evidence of DENV by using either NS1 antigen or polymerase chain reaction (PCR) assays. The serotype and genotype analyses were performed on a weekly basis to provide a timely update on the composition and distribution of DENV populations to facilitate the prioritizing of resource allocation for dengue control operations. The screening also included individual field-caught mosquitoes that were positive for DENV NS1 antigen. Viral RNA from mosquitoes was extracted as described previously [28]. DENV-positive sera and mosquito specimens were further analysed to determine the serotype of DENV by using a real time reverse-transcription PCR (RT-PCR) assay as described elsewhere [26]. At EHI, one of the public health laboratories in Singapore, all DENV-positive sera that failed serotype screening by the real time RT-PCR assay were subjected to a modified semi-nested conventional PCR assay [29, 30].

For the genotype surveillance, *E* gene of DENV was PCR amplified and sequenced using serotype-specific primers as described previously [29]. Nucleotide sequences were assembled using the Lasergene package version 8.0 (DNASTAR Inc., Madison, WI, USA). Contiguous sequences were aligned using BioEdit v7.0.5 software [31]. Phylogenetic analysis of *E* gene sequences was performed in MEGA6 program [32] using the maximum-likelihood method based on the general time reversible model with gamma distribution and invariant sites. The robustness of the original tree was tested with 1000 bootstrap replications.

### Entomological data collection

As part of active preventive surveillance, premise checks for larval breeding were undertaken by approximately 800 ground officers from the Environmental Public Health Operations, NEA, with an aim of checking every home and surrounding areas every three to six months. Such source reduction and vector surveillance efforts were enhanced in vicinities with high risk of DENV transmission, such as dengue clusters. *Aedes* immatures collected by field officers were morphologically identified to the species level at EHI. The number and type of breeding places as well as mosquito identification data was recorded in a common database for operational purposes. The mosquito identification data was also used for enforcement activities. In addition, adult *Aedes* population monitoring was conducted with about 3,000 Gravitraps at 34 sentinel locations weekly and within selected cluster areas on *ad hoc* basis. The design and deployment of Gravitraps have previously been described [24]. For all adult samples collected from clusters and sentinel locations, the abdomens of trapped *Aedes* mosquitoes were pooled into groups of five and screened for DENV by using a Dengue NS1 antigen assay as previously described [28].

### Data analysis

Incidence rates were calculated using all laboratory-confirmed cases reported to MOH based on the estimated mid-year population obtained from the Singapore Department of Statistics, Ministry of Trade and Industry. The house index and *Ae. aegypti* house index were calculated based on *Aedes* breeding data collected by ground officers through routine premise inspections. The *Ae. aegypti* house indices from 2012 to 2014 were compared by using the paired two sample t test in R software version 3.1.1 [33]. The Gravitrap *aegypti* index was calculated based on adult *Ae. aegypti* data generated through the Gravitrap sentinel system. The index expresses the percentage of Gravitraps that caught at least one *Ae. aeygpti* in a particular location. The relationship between the diagnostic rate and total number of reported cases was determined by calculating the Spearman’s Correlation Coefficient in R software version 3.1.1 [33]. The weekly national serotype data and weekly EHI genotype data from 2007 to 2014 were used to estimate the historical genotype proportions. In order to obtain smooth estimates of genotype proportions over time, we used Bayesian approach, assuming multinomial distribution of serotypes and genotypes, and an auto-correlated prior distribution for logarithm transformed proportions. We then sampled from the posterior distribution to get the Bayesian estimates of the weekly genotype proportions, which were used together with weekly national case count to calculate the weekly cases attributed to each genotype as well the cumulative case count. The analysis was done using R software version 3.1.1 [33]. Only the genotypes dominant during the epidemic years have been plotted in the graph.

The spatial distribution of dengue cases in Singapore was generated using the kernel density tool in the spatial analyst toolbox of ArcGIS 10.1 ArcMap software (ESRI, CA, USA) based on a search radius of 400 m. Case density values were classified into four classes of < 25^th^ (2 cases/km^2^), 25^th^-50^th^ (16-25 cases/km^2^), 51^st^-75^th^ (56-61 cases/km^2^) and more than 75^th^ (217 cases/km^2^) quantiles, using the quantile classification method, so that higher densities were shown in darker tones of pink and vice versa.

## Abbreviations

3P, people, public and private; DENV, Dengue virus; DHF, dengue hemorrhagic fever; DSS, dengue shock syndrome; E, Envelope; EHI, Environmental Health Institute; HDB, Housing Development Board; MOH, Ministry of Health; NEA, National Environment Agency; NS1, Non-structural protein 1; PCR, polymerase chain reaction; RNA, Ribose Nucleic Acid; RT-PCR, Reverse Transcription PCR.

## Endnotes

Not applicable.

## References

[1] Murray NE, Quam MB, Wilder-Smith A (2013). Epidemiology of dengue: past, present and future prospects. Clin Epidemiol.

[2] Bhatt S, Gething PW, Brady OJ, Messina JP, Farlow AW, Moyes CL, Drake JM, Brownstein JS, Hoen AG, Sankoh O (2013). The global distribution and burden of dengue. Nature.

[3] Calisher CH, Karabatsos N, Dalrymple JM, Shope RE, Porterfield JS, Westaway EG, Brandt WE (1989). Antigenic relationships between flaviviruses as determined by cross-neutralization tests with polyclonal antisera. J Gen Virol.

[4] Simmons CP, Farrar JJ, Nguyen VV, Wills B (2012). Dengue. N Engl J Med.

[5] Rico-Hesse R (1990). Molecular evolution and distribution of dengue viruses type 1 and 2 in nature. Virology.

[6] Goncalvez AP, Escalante AA, Pujol FH, Ludert JE, Tovar D, Salas RA, Liprandi F (2002). Diversity and evolution of the envelope gene of dengue virus type 1. Virology.

[7] Holmes EC, Twiddy SS (2003). The origin, emergence and evolutionary genetics of dengue virus. Infect Genet Evol.

[8] Gubler DJ (1998). Dengue and dengue hemorrhagic fever. Clin Microbiol Rev.

[9] WHO (2009). Dengue: Guidelines for diagnosis, treatment, prevention and control.

[10] Chan KL, Ng SK, Chew LM (1977). The 1973 dengue haemorrhagic fever outbreak in Singapore and its control. Singapore Med J.

[11] Goh KT (1997). Dengue--a re-emerging infectious disease in Singapore. Ann Acad Med Singapore.

[12] Chan YC, Ho BC, Chan KL (1971). Aedes aegypti (L.) and Aedes albopictus (Skuse) in Singapore City. 5. Observations in relation to dengue haemorrhagic fever. Bull World Health Organ.

[13] Goh KT (1995). Changing epidemiology of dengue in Singapore. Lancet.

[14] Koh BK, Ng LC, Kita Y, Tang CS, Ang LW, Wong KY, James L, Goh KT (2008). The 2005 dengue epidemic in Singapore: epidemiology, prevention and control. Ann Acad Med Singapore.

[15] Lee KS, Lai YL, Lo S, Barkham T, Aw P, Ooi PL, Tai JC, Hibberd M, Johansson P, Khoo SP, Ng LC (2010). Dengue Virus Surveillance for Early Warning, Singapore. Emerg Infect Dis.

[16] Ler TS, Ang LW, Yap GS, Ng LC, Tai JC, James L, Goh KT (2011). Epidemiological characteristics of the 2005 and 2007 dengue epidemics in Singapore - similarities and distinctions. Western Pac Surveill Response J.

[17] Ang LW, Cutter J, James L, Goh KT (2015). Seroepidemiology of dengue virus infection in the adult population in tropical Singapore. Epidemiol Infect.

[18] Yew YW, Ye T, Ang LW, Ng LC, Yap G, James L, Chew SK, Goh KT (2009). Seroepidemiology of dengue virus infection among adults in Singapore. Ann Acad Med Singapore.

[19] Goh KT, Yamazaki S (1987). Serological survey on dengue virus infection in Singapore. Trans R Soc Trop Med Hyg.

[20] Low SL, Lam S, Wong WY, Teo D, Ng LC, Tan LK (2015). Dengue Seroprevalence of Healthy Adults in Singapore: Serosurvey Among Blood Donors, 2009. Am J Trop Med Hyg.

[21] Lee KS, Lo S, Tan SS, Chua R, Tan LK, Xu H, Ng LC (2012). Dengue virus surveillance in Singapore reveals high viral diversity through multiple introductions and in situ evolution. Infect Genet Evol.

[22] Ng LC, Chem YK, Koo C, Mudin RN, Amin FM, Lee KS, Kheong CC (2015). 2013 Dengue Outbreaks in Singapore and Malaysia Caused by Different Viral Strains. Am J Trop Med Hyg.

[23] Shi Y, Liu X, Kok SY, Rajarethinam J, Liang S, Yap G, Chong CS, Lee KS, Tan SS, Chin CK, et al. Three-Month Real-Time Dengue Forecast Models: An Early Warning System for Outbreak Alerts and Policy Decision Support in Singapore. Environmental health perspectives. 2015. doi:10.1289/ehp.1509981. 10.1289/ehp.1509981 PMC5010413 26662617

[24] Lee C, Vythilingam I, Chong CS, Abdul Razak MA, Tan CH, Liew C, Pok KY, Ng LC (2013). Gravitraps for management of dengue clusters in Singapore. Am J Trop Med Hyg.

[25] Ng LC, Vythilingum, I (Ed.). Vectors of Flaviviruses and Strategies for Control, in Molecular Virology and Control of Flaviviruses. Poole, United Kingdom: Caister Academic Press; 2011.

[26] Lai YL, Chung YK, Tan HC, Yap HF, Yap G, Ooi EE, Ng LC (2007). Cost-effective real-time reverse transcriptase PCR (RT-PCR) to screen for Dengue virus followed by rapid single-tube multiplex RT-PCR for serotyping of the virus. J Clin Microbiol.

[27] Pok KY, Lai YL, Sng J, Ng LC. Evaluation of Nonstructural 1 Antigen Assays for the Diagnosis and Surveillance of Dengue in Singapore. Vector Borne Zoonotic Dis. 2010. 10.1089/vbz.2008.0176 PMC2992696 20426686

[28] Tan CH, Wong PS, Li MZ, Vythilingam I, Ng LC (2011). Evaluation of the Dengue NS1 Ag Strip(R) for detection of dengue virus antigen in Aedes aegypti (Diptera: Culicidae). Vector Borne Zoonotic Dis.

[29] Koo C, Nasir A, Hapuarachchi HC, Lee KS, Hasan Z, Ng LC, Khan E (2013). Evolution and heterogeneity of multiple serotypes of Dengue virus in Pakistan, 2006-2011. Virol J.

[30] Lanciotti RS, Calisher CH, Gubler DJ, Chang GJ, Vorndam AV (1992). Rapid detection and typing of dengue viruses from clinical samples by using reverse transcriptase-polymerase chain reaction. J Clin Microbiol.

[31] Hall TA (1999). BioEdit: a user-friendly biological sequence alignment editor and analysis program for Windows 95/98/NT. Nucl Acid Symp Ser.

[32] Tamura K, Stecher G, Peterson D, Filipski A, Kumar S (2013). MEGA6: Molecular Evolutionary Genetics Analysis version 6.0. Mol Biol Evol.

[33] RCoreTeam. R: A language and environment for statistical computing. Vienna, Austria: R Foundation for Statistical Computing; 2014.

